# Kinetics of the West Nile virus induced transcripts of selected cytokines and Toll-like receptors in equine peripheral blood mononuclear cells

**DOI:** 10.1186/s13567-016-0347-8

**Published:** 2016-06-07

**Authors:** Muhammad Jasim Uddin, Willy W. Suen, Angela Bosco-Lauth, Airn-Elizabeth Hartwig, Roy A. Hall, Richard A. Bowen, Helle Bielefeldt-Ohmann

**Affiliations:** School of Veterinary Science, The University of Queensland, Gatton, QLD Australia; Department of Biomedical Sciences, Animal Reproduction and Biotechnology Laboratory, Colorado State University, Fort Collins, USA; School of Chemistry and Molecular Biosciences, The University of Queensland, St Lucia, QLD Australia; Australian Infectious Diseases Research Centre, The University of Queensland, St Lucia, QLD Australia

## Abstract

West Nile virus (WNV) is one of the most common causes of epidemic viral encephalitis in horses worldwide. Peripheral blood mononuclear cells (PBMCs) are amongst the first to encounter the virus following a mosquito bite. This study aimed to elucidate the transcription kinetics of cytokine, Toll-like receptor (TLRs) and TLRs-associated genes following WNV challenge of equine PBMCs. PBMCs were challenged with an Australian strain of WNV (WNV_NSW2011_) and transcriptomes were quantified at 2, 6, 12 and 24 h post-infection (pi) using qRT-PCR. Type I and II interferons (IFNα, β and γ) mRNA transcription increased following WNV exposure, as did the transcripts for IL1α, IL1β, IL6, IL8, and IL22, but with slightly varying kinetics. TLR1, 3, 5, 7-9 transcripts were also upregulated in equine PBMCsin response to WNV challenge, as were those for MyD88, NF-κB, TRAF3, STAT1 and 2, IRF3 and 7, ISG15, as well as SOCS1 and 3 compared to the control cells. Expression of selected genes in the draining lymph node, spleen and brain (medulla oblongata) of experimentally infected horses was also assessed and transcription of most of these genes was also upregulated here. Although qRT-PCR detected higher viral RNA at 24 h pi compared to 6 h pi, the virus did not replicate productively in equine PBMCs. The up-regulation of gene-transcription for selected cytokines, IFNs, TLRs and TLRs-associated molecules suggests their involvement in virus recognition and control of WNV infection in the horse.

**Electronic supplementary material:**

The online version of this article (doi:10.1186/s13567-016-0347-8) contains supplementary material, which is available to authorized users.

## Introduction

West Nile virus (WNV), a mosquito-borne flavivirus, is widely distributed throughout Africa, the Middle East, Asia, Southern Europe, the Americas and Australia [1]. Since the first isolation of the Australian strain of WNV—Kunjin strain (WNV_KUN_) in 1960 in North Queensland [2], it has been found to be endemic in Australia [3]. WNV_KUN_ belongs to lineage 1 of WNV, which also contains the highly pathogenic, neuroinvasive New York 99 strain (WNV_NY99_) [4]. From 1999 to 2014, a total of 38 259 humans infections with 1654 deaths and more than 20 000 equine deaths of WNV disease have been reported in the USA [5]. Relative to the WNV_NY99_ strain, WNV_KUN_ exhibits much reduced virulence in humans, animals, and birds. In early 2011 following extensive flooding in the Murray-Darling River basin and other inland river systems of South-East Australia, an unprecedented outbreak of equine encephalitis occurred in south-eastern Australia involving more than 1000 horses [4]. Only a few human clinical cases occurred at the time and none were fatal. Genomic sequencing of viruses isolated from affected horses and mosquitoes in the 2011 outbreak revealed the aetiological agent to be a variant strain of WNV, most closely related to WNV_KUN_ and indicating local origin [4]. The new strain was named WNV_NSW2011_. At the genetic level, WNV_NSW2011_ is more than 98% homologous in amino acid sequence to WNV_NY99_ [4]. Recently it was found that WNV remains a potential threat to human and animal health in Australia [6], however, despite its importance much remains unknown regarding the host-virus relationship in the target species, humans and equines [7].

To survive virus infection, the host must recognise invasion and develop an effective antiviral immune response. This response is initiated in infected cells after detection of virus by specific, conserved host molecules, such as Toll-like receptors (TLRs) which trigger signalling cascades that induce the activation of transcription factor NF-κB, IFN regulatory factors (IRFs), including IFNs and hundreds of different IFN-stimulated effector genes (ISGs) [8]. The involvement of TLR3 [9], 7 [10] and 9 [11] in WNV infection has been widely studied in mice and humans. MyD88, NF-κB, STAT, ISG15, IRF7and 9 [12–16] are crucially involved in the recognition and induction of host immune response to WNV. As a result of successful recognition of WNV by TLRs and associated genes, cytokine andIFN cascades are activated to prevent the early stages of WNV replication [17]. The ISG products include antiviral effector molecules and immunomodulatory cytokines that serve to restrict virus replication and modulate the adaptive immune response [15]. However, these signalling pathways have been studied predominantly in mice (reviewed in [8]), with only limited studies in horses [18]. WNV typically produce severe encephalitis and death in mice (reviewed in [19]), while most WNV infections in humans and horses are subclinical [20–22]. The transcriptome profile in small animal models may therefore not reflect what happens in the majority of human and equine infections, thus warranting alternative models [23]. Furthermore, while WNV_NY99_ causes severe neuropathology and death in mice, WNV_KUN_ mostly causes only subclinical infection in horses and rabbits [23].

Identification of molecules that may be involved in limiting virus growth and contributing to host survival is therefore crucial to understand the host-pathogen interactions of subclinical infection. Therefore, we hypothesised that WNV_KUN_ induces a pattern of innate immune responses, which might help to elucidate the host-pathogen interactions of intermediate virulent flaviviruses [23–25]. Furthermore, the virus in most natural cases of equine WNV encephalitis is undetectable in the central nervous system (CNS) at the time of clinical symptoms appearing [20, 21] suggesting that an early innate immune response may bean important factor for limiting CNS infection and survival of horses [26]. Peripheral blood mononuclear cells (PBMCs) are amongst the first immune components to encounter WNV following a mosquito bite. It has been suggested that the equine PBMCs may serve as target cells for North American strains of WNV and play a role in subsequent viral dissemination [27]. Therefore, the current study aimed at assessing the kinetics of innate immune molecule transcripts activated at early time points following WNV_NSW2011_ infection of equine PBMCs. For this purposes, WNV_NSW2011_ stimulated equine PBMCs were cultured for 24 h and gene transcripts, which might be involved in the innate immune response to virus, were quantified.

## Materials and methods

### Preparation of WNV

The WNV_NSW2011_ strain used in this study has previously been characterized and described in detail [4]. Two additional passages of the virus were performed in C6/36 mosquito (*Aedes albopictus*) cells cultured in RPMI-1640 media with 2% foetal bovine serum (FBS) at 28 °C without CO_2_ [28]. The virus stock was stored at −80 °C. The titre of the stock was quantified using standard plaque assay on Vero cells, as described previously [28]. Briefly, samples were serially diluted in PBS in the range 1:10^3^ to 1:10^9^. Three hundred microliters (300 µL) of sample was added to each well of a 6-well plate with a confluent monolayer of Vero cells. The plates were incubated for 2 h at 37 °C, then 2 mL of overlay was added to each well (final concentration = 0.5% agar with 1× DMEM, 2% FBS, l-glutamine [2 nM], penicillin [50 U/mL] and streptomycin [50 µg/mL]). Plates were incubated in a 37 °C with 5% CO_2_ incubator. At 4 days post-infection (pi), 2 mL of 10% formaldehyde was added and incubated for 30 min at room temperature to fix the plate. The gel overlay was removed and 1 mL of 0.2% crystal violet was added and incubated for 30 min. Plaques were enumerated using a light box. The WNV_NSW2011_ was diluted to 1 × 10^6^ PFU/50 µL for use in PBMCs infection in vitro experiments.

### PBMC isolation, culture and challenged with viruses

The studies were approved by the University of Queensland Animal Ethics Committee (AEC nos. SVS/306/11/VAXINE and SVS/298/13/VAXINE), and the Animal Use and Care Committee of Colorado State University (Approval: 12-3837A), and carried out in accordance with the NHMRC and NIH guidelines for ethical use of animals in research. EDTA-stabilized blood samples were collected from three healthy WNV seronegative horses (*n* = 3) [29]. Blood was collected from the jugular vein of horses into EDTA-coated collection tubes (Vacuette^®^, Greiner Bio One, Australia). The PBMCs were isolated from the blood using Ficoll-Histopaque (Sigma) as described previously [25, 30]. The viability of the purified PBMCs was assessed using the trypan blue exclusion method and was always greater than 90%. The cells were counted using a haemocytometer and the concentration was adjusted to 1 × 10^6^ cells/0.5 mL in RPMI-1460 supplemented with l-glutamine (2 nM), streptomycin (50 µg/mL) and penicillin (50 U/mL), and 10% FBS [25, 30]. The cells were cultured in a 24-well cell culture plate (Costar Corning, the Netherlands) seeded with 500 µL cell suspension per well and at 37 °C with 5% CO_2_. Remaining PBMCs (fresh-isolated PBMCs) were pelleted and kept at −80 °C for RNA isolation. After 1 h of seeding, the PBMCs were challenged by adding 50 µL of WNV_NSW2011_ stock to each well to give a final multiplicity of infection (MOI) of one [7, 25, 31]. Additional 450 µL medium was added to each well to make up the final volume 1 mL. One well of PBMCs per animal (*n* = 3) was harvested at 2 h, 6 h, 12 h and 24 h after virus infection. The harvested cells (WNV-stimulated PBMCs) were pelleted and kept at −80 °C until subjected to RNA isolation. For complete harvest of adherent PBMCs, detachment with lidocaine HCl (12 mM) was performed [32]. To ensure the complete harvesting of cells, wells were checked using an inverted microscope. Duplicate wells of uninfected cells (mock-inoculated PBMCs) were harvested and treated in a similar manner at each time point.

### Experimentally horse infection with WNV

Three horses (all quarterstock horses, 1–2 years old; one gelding and two females) seronegative for WNV were challenged by intradermal inoculation of 3 × 10^4^ PFU of WNV_NSW2011_. Horses were euthanatized 12 days pi [33]. Samples from CNS (medulla oblongata), spleen and lymph nodes (the injection-site draining pre-scapular) were collected at necropsy for RNA isolation, histopathology assessment and immunohistochemistry.

### RNA isolation, cDNA synthesis and transcriptome quantification

Total RNA was isolated from PBMCs using miRN easy RNA isolation kit (Qiagen Pty Ltd, Australia) following the manufacturer’s instructions. Total RNA from tissues from the experimentally infected horses was isolated using MagMAX-96 Viral RNA Isolation Kit (Life Technologies). On column RNA clean-up was performed using DNase I digestion kit (Qiagen) following the manufacturer’s protocol. The quantity and quality of RNA was measured using NanoDrop 1000 spectrophotometer (Thermo Scientific, Australia). The isolated RNA was kept at −80 °C for further use. cDNA synthesis was performed using ‘qScript cDNA synthesis kit’ (cat. No: 95047-100, Quanta Biosciences) following the manufacturer’s protocol and stored at −20 °C until employed in transcriptome analysis using quantitative real time PCR (qRT-PCR). Primers for cytokines, TLRs and downstream genes, apoptosis and oxidative stress related genes, and two house-keeping genes (*ACTB* and *GAPDH*) were designed from FASTA products of the GenBank mRNA sequences for *Equus caballus* using the Primer3 program [34]. The WNV_KUN_ specific primers were described earlier [35]. Details of the primers are given in Table 1. To quantify the mRNA expression for target and reference genes, qRT-PCR was performed using the Rotor Gene Corbett 6000 quantitative real-time PCR system (Qiagen).

**Table 1:** **List of primers used in this study**

Gene	Primer set^a^	Amplicon size (bp)	Gene Bank accession number
ACTB	F: TGAGCGCAAGTACTCCGTAT R: TCCTGCTTGCTGATCCACAT	96	NM_001081838.1
GAPDH	F: GGTGAAGGTCGGAGTAAACG^b^ R: AATGAAGGGGTCATTGATGG	106	NM_001163856.1
IFNα	F: TCTTGATGCTCCTGGGACAA R: AACTGGTTGCCGTCAAACAC	103	NM_001099441.1
IFNβ	F: ACCATTCTGCGCCTGAAGAA R: AGGCCAAGTTCCTGAGCATT	115	NM_001099440.1
IFNγ	F: TTAACAGCAGCACCAGCAAG R: TTTGCGCTGGACCTTCAGAT	80	NM_001081949.1
TNFα	F: AAGCCTGTAGCCCATGTTGT R: TCTGTCAGCTTCACGCCATT	104	NM_001081819.1
IL1α	F: ATGAGGATCGTCAACCACCA R: TTCACTGCGTCGTCCAGATT	119	NM_001082500.1
IL1β	F: TCAAAGTCAGCCTGGTGGAA R: TTGCTCATCAGAAGCTGGGT	80	NM_001082526.1
IL6	F: TCTTCACAAGCACCGTCACT R: AGTGGTCCATTTGAGGTGGT	118	NM_001082496.1
IL8	F: TCAAGACGCACTCCAAACCT R: AGCTCCGTTGACGAGCTTTA	113	NM_001083951.1
IL12	F: ATCGTGGTGGATGCTGTTCA R: TAATGGCTTCAGCTGCAGGT	111	NM_001082516.1
IL22	F: TTCCAGCCTTACATGCAGGA R: TGCTGGTCATCACCCTCAAT	89	XM_001491754.3
PTX3	F: TCTGTGCAGCACCTGGAATT R: ACAACATGACCTGTGGCCAT	99	XM_005602088.1
CXCL10	F: GCACGCTGTACCTGCATTAA R: TGGCAATGATCTCAACACGT	112	NM_001114940.1
TLR1	F: TGTGGTGCCTTTAACAGCCT R: TTCTGGCAGCTCTGGAAGAA	89	NM_001256899.1
TLR2	F: TGCGAATCCTGAAAGTGGGA R: ACCGCTGGAGATTTGTAGCA	111	NM_001081796.1
TLR3	F: CCTGCAACAAGTGTGCTGTT R: TGGGTGAGATTCAACACCGT	109	NM_001081798.1
TLR4	F: AGCACTTCATTCAGAGCCGA R: TGAAGATGATGCCAGCACGA	90	NM_001099769.1
TLR5	F: TCCATGGAGGGTTGTGATGA^c^ R: CCCCGGAACTTTGTGACAAT		
TLR6	F: CTGGCAAGAGCATTGTGGAA R: ATGGCACCACTCACTCTGAA	101	NM_001257142.1
TLR7	F: TTGAGTGGCCAAGAAACCCA R: ACCGTCTCTTTGAACACCTGA	109	NM_001081771.2
TLR8	F: TGGGCAAGTACGTGACAGAA R: GTGCCTTGCCATTGTGGTTT	120	NM_001111301.1
TLR9	F: AGCATCTTCGCACAAGACCT R: AGGTGGTGCAACATAGGCAT	116	NM_001081790.1
TLR10	F: TCAAACTCTCCTGCAGCCAA R: AGTTGTTTGACCGCCGAGAT	64	XM_005608830.1
MyD88	F: TGTGTTCCACTTGCCTCTCA R: ACGCAGGACATGAGATGTGA	97	XM_001488549.4
TRAF3	F: ACGACCAGATGCTGAGTGTT R: CCAAATCAGCACGCCATTGT	98	XM_001490000.3
STST1	F: TGCCTTGATCAGCTGCAGAA R: TGCTCCAGTTCCTCAAGCTT	92	XM_005601647.1
STST2	F: TGGAAAGTCCAGCAGCAGAA R: TGCTCCAGCTGTGAACCAAT	87	XM_001504841.2
IRF3	F: AAGGTTGTTCCCACATGCCT R: GTGGCTGTTGGAAATGTGCA	102	XM_005596407.1
IRF7	F: TGTTGTCACACTCATCGCCA R: ACGTGTGGATTGAGGGCTTA	80	XM_005598388.1
NFkB	F: ACCAGTGTCATCGAGCAGAT R: TGCAGGTGGTTGGTGAGATT	80	XM_001916418.3
ISG15	F: CAGTTCTGGCTGACTTTCGA R: CAGGCGCAAGTTCATGTACA	102	XM_005607605.1
SOCS1	F: ATTTAACTGTGTCTGGCGCC R: CAACCCCTGGTTTGTGCAAA	81	XM_005615004.1
SOCS3	F: TCTCCAACATCTCTGTCGGA R: TTAAAGCGGGGCATCGTACT	115	NM_001123379.1
Caspase 3	F: ATGCAGCAAACCTCAGGGAA R: ATGCCACAATTTCTTCGCCG	87	NM_001163961.1
HMOX1	F: TGGCTTCTTCCTTTGGGCAT R: TGCTTGTTGGTTGGGGAAGA	108	XM_005606579.1
WNV_NSW2011_	F: AACCCCAGTGGAGAAGTGGA^d^ R: TCAGGCTGCCACACCAAA	70	D00246

^a^Annealing temperature was 60 °C for all the primer sets.

^b^Primer set is adopted from Beckman et al. [54].

^c^Kwon S et al. [55].

^d^Pyke et al. [35].

A two-step qRT-PCR was performed using Rotor-Gene SYBR Green PCR Kit (Qiagen). Each run contained cDNA samples and a no-template control. qRT-PCR was set up using 1 μL of cDNA template, 10.5 μL of deionized RNase free water, 0.5 μM of upstream and downstream primers, and 12.5 μL of 2× Rotor-Gene SYBR Green PCR Master Mix (MM) (Qiagen) in a total reaction volume of 25 μL. The cycling conditions included an initial PCR activation step (5 min at 95 °C), and a two-step cycling protocol, with a denaturation step and a combined annealing/extension step. The two-step thermal cycling conditions were 5 s at 95 °C followed by 10 s at 60 °C (40 cycles). An amplification-based auto-threshold (Rotor-Gene Q Series Software, Qiagen) and adaptive baseline were selected as algorithms. Melting curve analysis was performed to detect the specificity of the PCR reaction. Each sample was run twice and the average value was used as expression value. Gene-specific expression was measured as relative to the geometric mean of the expression of two housekeeping genes (GAPDH and ACTB) (Table 1). The delta ${\text{Ct }}\left( {\Delta {\text{Ct}}} \right) \, \left[ {\Delta {\text{Ct}} = {\text{Ct}}_{\text{target}} - {\text{Ct}}_{\text{house keeping genes}} } \right]$ values were calculated as the difference between target gene and reference genes and expression was calculated as 2^(−∆∆Ct)^ [36]. Although the widely accepted delta delta Ct method has been used to calculate the relative gene expression, it is important to determine the PCR efficiency for each transcript.

**Figure 1:**
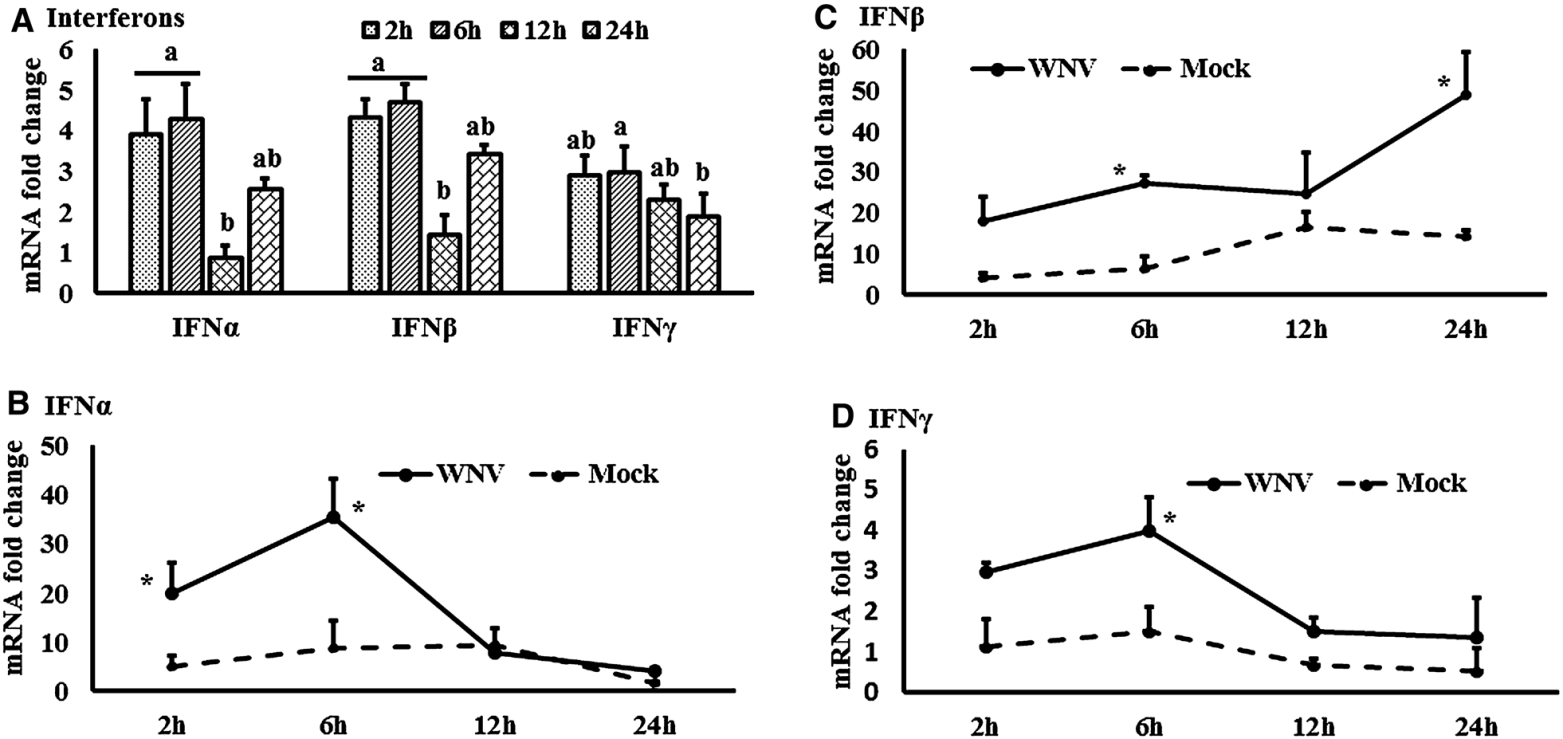
**Expression kinetics of interferons mRNA.** **A** Interferons expression in fold change. The ∆∆Ct $\left[ {\Delta \Delta {\text{Ct}} = \Delta {\text{Ct}}_{\text{WNV}} - \Delta {\text{Ct}}_{\text{mock}} } \right]$ values were calculated by subtracting the ∆Ct of genes in mock-inoculated PBMCs. The bar graph showed the expression of genes in WNV-infected PBMCs over mock-inoculated PBMCs (fold change: the normalised expression value of a gene in WNV-stimulated cells/the normalised expression value of a gene in mock-inoculated cells). Bars without common superscripts (**A**, **B**) denote statistical significant difference among time points (*P* < 0.05). **B**–**D** Relative expression of interferons mRNA, accounting for the effects of culture conditions on gene transcription in WNV- and mock-inoculated equine PBMCs. To compare the normalised expression of interferon genes from PBMCs harvested at each time point to their respective expression levels before either WNV- or mock- inoculation, the ∆∆Ct values were calculated by subtracting ∆Ct of genes in fresh-isolated PBMCs from the ∆Ct of genes in WNV- or mock- inoculated PBMCs at each time-point (for WNV-stimulated PBMCs, $\Delta \Delta {\text{Ct}}_{\text{WNV}} = \Delta {\text{Ct}}_{\text{WNV}} - \Delta {\text{Ct}}_{\text{fresh}}$; and for mock-inoculated PBMCs, $\Delta \Delta {\text{Ct}}_{\text{mock}} = \Delta {\text{Ct}}_{\text{mock}} - \Delta {\text{Ct}}_{\text{fresh}}$). *P < 0.05.

**Figure 2:**
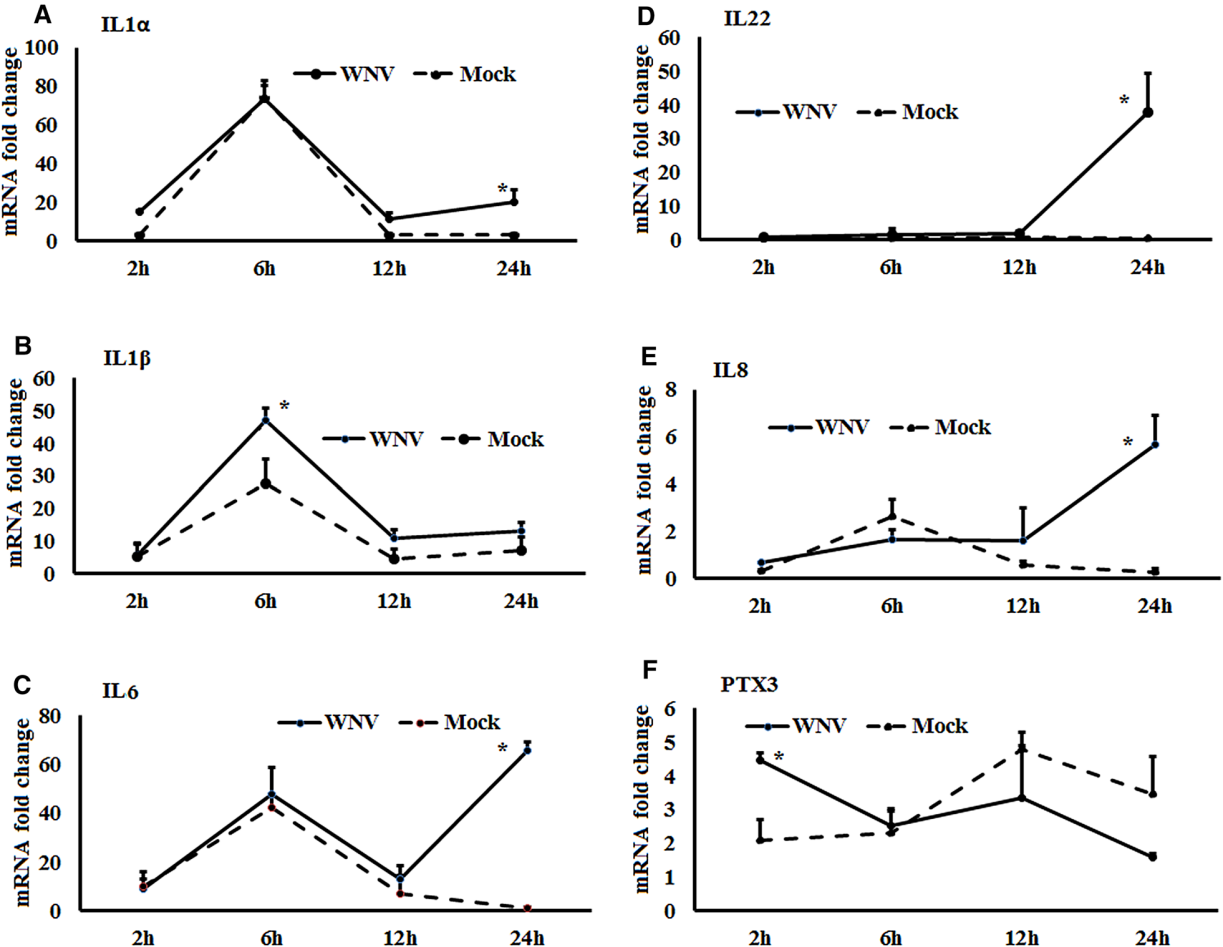
**Expression kinetics of cytokines mRNA.** **A**–**F** Relative expression of cytokines mRNA, accounting for the effects of culture conditions on gene transcription in WNV- and mock-inoculated equine PBMCs. To compare the normalised expression of cytokine genes from PBMCs harvested at each time point to their respective expression levels before either WNV- or mock- inoculation, the ∆∆Ct values were calculated by subtracting ∆Ct of genes in fresh-isolated PBMCs from the ∆Ct of genes in WNV- or mock- inoculated PBMCs at each time-point (for WNV-stimulated PBMCs, $\Delta \Delta {\text{Ct}}_{\text{WNV}} = \Delta {\text{Ct}}_{\text{WNV}} - \Delta {\text{Ct}}_{\text{fresh}}$; and for mock-inoculated PBMCs, $\Delta \Delta {\text{Ct}}_{\text{mock}} = \Delta {\text{Ct}}_{\text{mock}} - \Delta {\text{Ct}}_{\text{fresh}}$). *P < 0.05.

**Figure 3:**
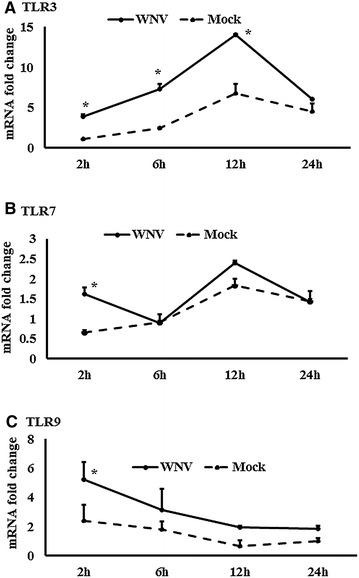
**Expression kinetics of TLRs mRNA.** **A**–**C** Relative expression of TLRs mRNA, accounting for the effects of culture conditions on gene transcription in WNV- and mock-inoculated equine PBMCs. To compare the normalised expression of TLRs genes from PBMCs harvested at each time point to their respective expression levels before either WNV- or mock- inoculation, the ∆∆Ct values were calculated by subtracting ∆Ct of genes in fresh-isolated PBMCs from the ∆Ct of genes in WNV- or mock- inoculated PBMCs at each time-point (for WNV-stimulated PBMCs, $\Delta \Delta {\text{Ct}}_{\text{WNV}} = \Delta {\text{Ct}}_{\text{WNV}} - \Delta {\text{Ct}}_{\text{fresh}}$; and for mock-inoculated PBMCs, $\Delta \Delta {\text{Ct}}_{\text{mock}} = \Delta {\text{Ct}}_{\text{mock}} - \Delta {\text{Ct}}_{\text{fresh}}$). *P < 0.05.

**Figure 4:**
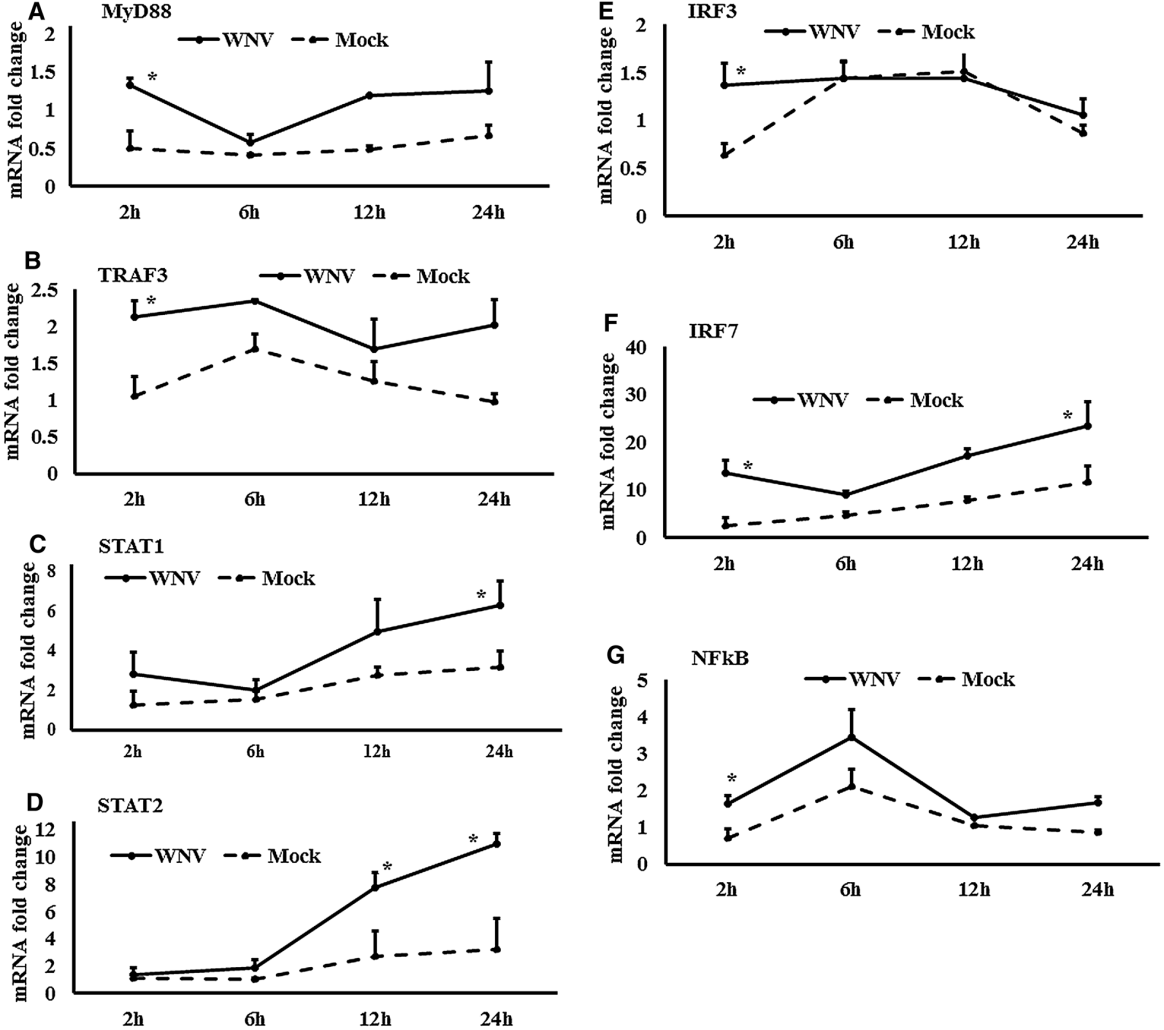
**Expression kinetics of TLR-associated genes transcriptions.** **A**–**G** Relative expression of mRNA for TLRs-associated downstream genes, accounting for the effects of culture conditions on gene transcription in WNV- and mock-inoculated equine PBMCs. To compare the normalised expression of TLRs-associated genes from PBMCs harvested at each time point to their respective expression levels before either WNV- or mock- inoculation, the ∆∆Ct values were calculated by subtracting ∆Ct of genes in fresh-isolated PBMCs from the ∆Ct of genes in WNV- or mock- inoculated PBMCs at each time-point (for WNV-stimulated PBMCs, $\Delta \Delta {\text{Ct}}_{\text{WNV}} = \Delta {\text{Ct}}_{\text{WNV}} - \Delta {\text{Ct}}_{\text{fresh}}$; and for mock-inoculated PBMCs, $\Delta \Delta {\text{Ct}}_{\text{mock}} = \Delta {\text{Ct}}_{\text{mock}} - \Delta {\text{Ct}}_{\text{fresh}}$). *P < 0.05.

**Figure 5:**
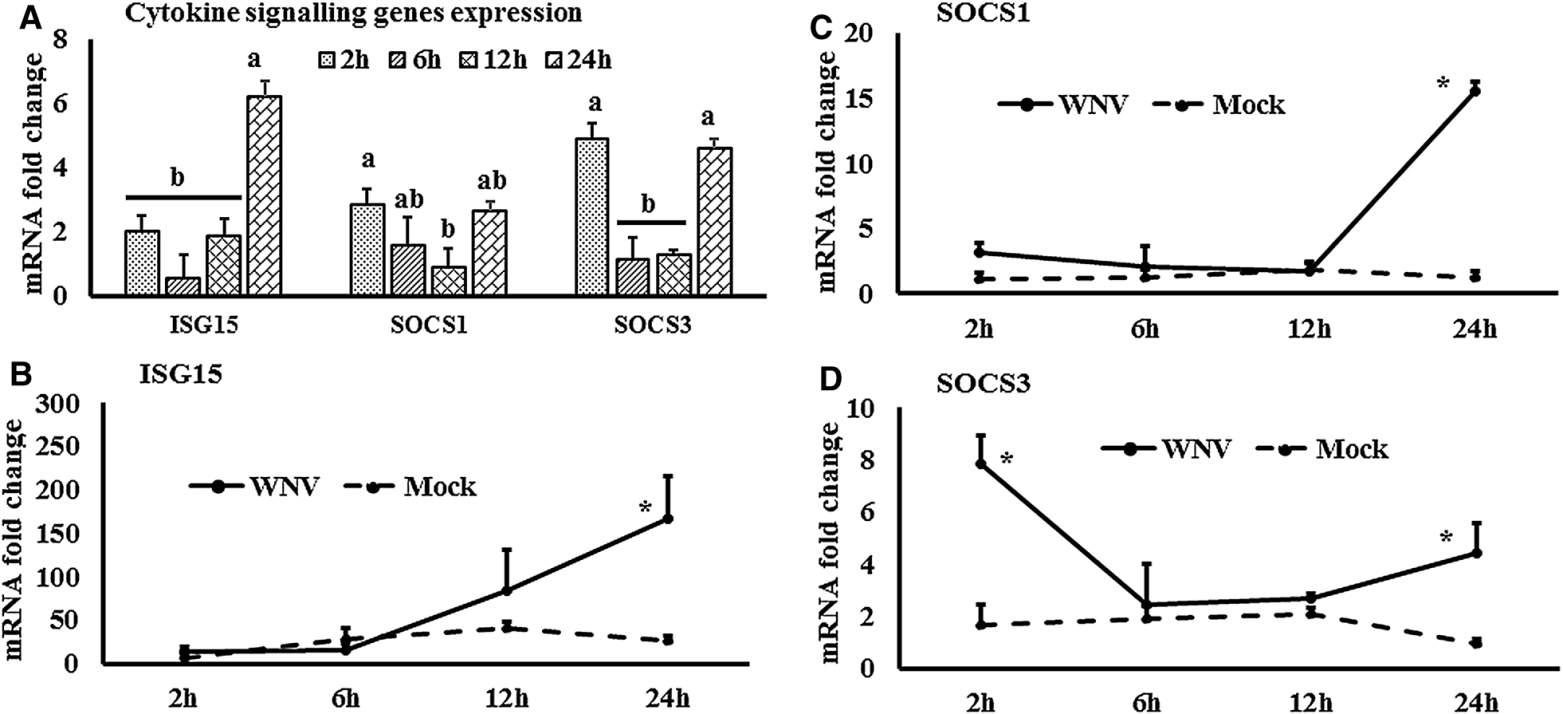
**Expression kinetics of ISG15 and SOCS mRNA.** **A** ISG and SOCS mRNA expression in fold change. The ∆∆Ct $\left[ {\Delta \Delta {\text{Ct}} = \Delta {\text{Ct}}_{\text{WNV}} - \Delta {\text{Ct}}_{\text{mock}} } \right]$ values were calculated by subtracting the ∆Ct of genes in mock-inoculated PBMCs. The bar graph showed the expression of genes in WNV-infected PBMCs over mock-inoculated PBMCs (fold change: the normalised expression value of a gene in WNV-stimulated cells/the normalised expression value of a gene in mock-inoculated cells). Bars without common superscripts (**A**, **B**) denote statistical significant difference among time points (*P* < 0.05). **B**–**D** Relative expression of ISG15 and SCOS mRNA, accounting for the effects of culture conditions on gene transcription in WNV- and mock-inoculated equine PBMCs. To compare the normalised expression of genes from PBMCs harvested at each time point to their respective expression levels before either WNV- or mock- inoculation, the ∆∆Ct values were calculated by subtracting ∆Ct of genes in fresh-isolated PBMCs from the ∆Ct of genes in WNV- or mock- inoculated PBMCs at each time-point (for WNV-stimulated PBMCs, $\Delta \Delta {\text{Ct}}_{\text{WNV}} = \Delta {\text{Ct}}_{\text{WNV}} - \Delta {\text{Ct}}_{\text{fresh}}$; and for mock-inoculated PBMCs, $\Delta \Delta {\text{Ct}}_{\text{mock}} = \Delta {\text{Ct}}_{\text{mock}} - \Delta {\text{Ct}}_{\text{fresh}}$). *P < 0.05.

**Figure 6:**
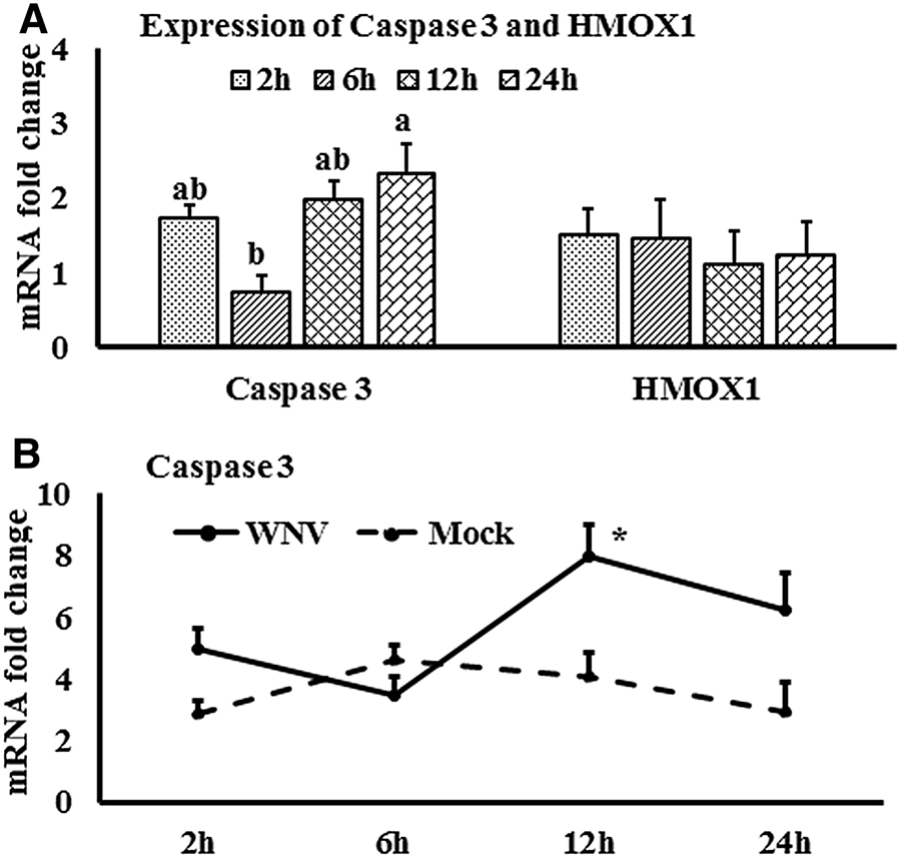
**Expression kinetics of Caspase 3 and HMOX1 mRNA.** **A** Caspase 3 and HMOX1 mRNA expression in fold change. The ∆∆Ct $\left[ {\Delta \Delta {\text{Ct}} = \Delta {\text{Ct}}_{\text{WNV}} - \Delta {\text{Ct}}_{\text{mock}} } \right]$ values were calculated by subtracting the ∆Ct of genes in mock-inoculated PBMCs. The bar graph showed the expression of genes in WNV-infected PBMCs over mock-inoculated PBMCs (fold change: the normalised expression value of a gene in WNV-stimulated cells/the normalised expression value of a gene in mock-inoculated cells). Bars without common superscripts (**A**, **B**) denote statistical significant difference among time points (*P* < 0.05). **B** Relative expression of caspase 3mRNA, accounting for the effects of culture conditions on gene transcription in WNV- and mock-inoculated equine PBMCs. To compare the normalised expression of caspase 3 gene from PBMCs harvested at each time point to their respective expression levels before either WNV- or mock- inoculation, the ∆∆Ct value were calculated by subtracting ∆Ct of gene in fresh-isolated PBMCs from the ∆Ct of gene in WNV- or mock- inoculated PBMCsat each time-point (for WNV-stimulated PBMCs, $\Delta \Delta {\text{Ct}}_{\text{WNV}} = \Delta {\text{Ct}}_{\text{WNV}} - \Delta {\text{Ct}}_{\text{fresh}}$; and for mock-inoculated PBMCs, $\Delta \Delta {\text{Ct}}_{\text{mock}} = \Delta {\text{Ct}}_{\text{mock}} - \Delta {\text{Ct}}_{\text{fresh}}$). *P < 0.05.

**Figure 7:**
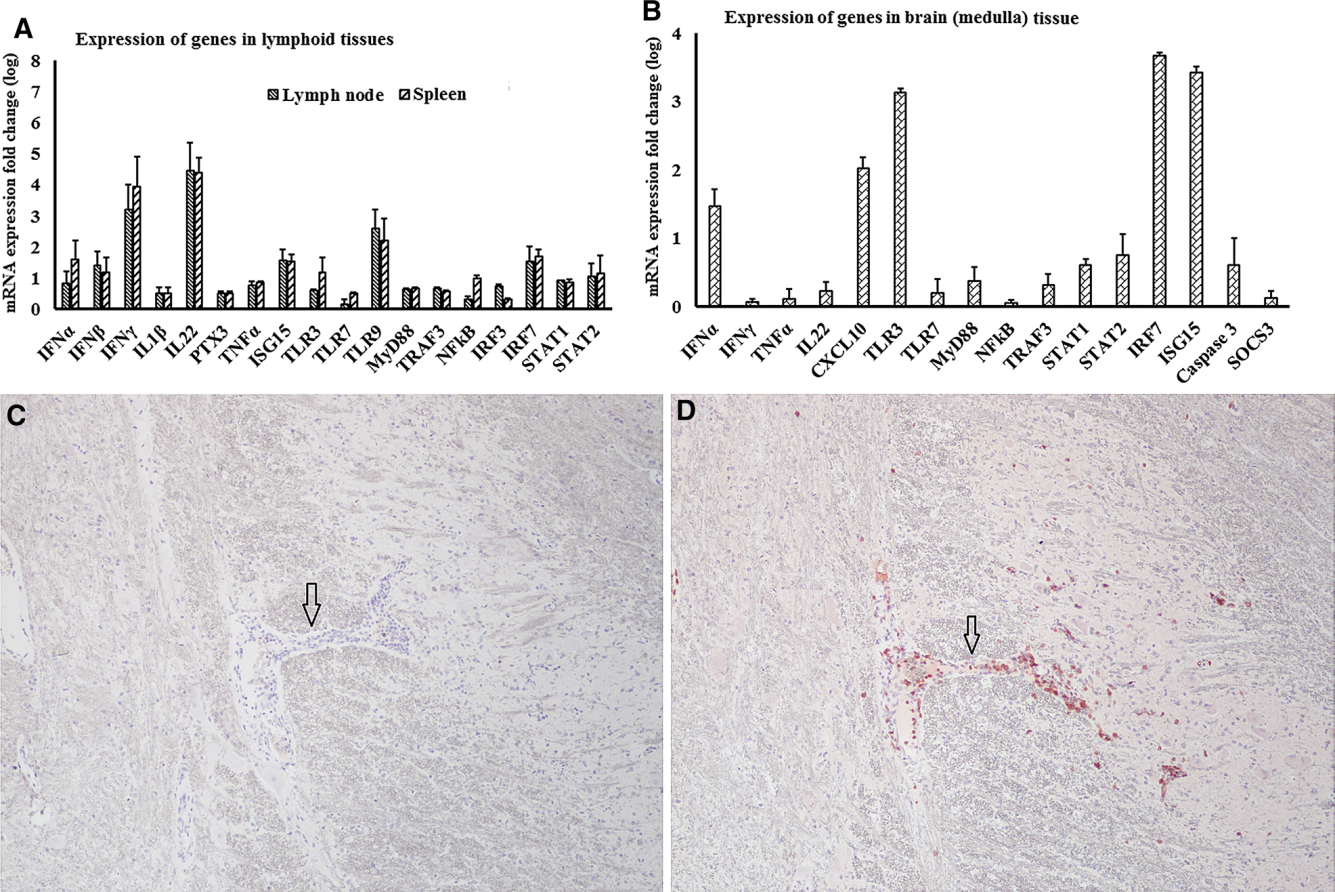
**mRNA quantification and viral antigen detection in tissues from WNV-infected horses.** **A**, **B** Quantification of selected genes transcripts in tissues from experimentally infected horses. **A** lymphoid tissues (draining lymph node, spleen) and (**B**) brain in fold change. To compare the gene expression between WNV_NSW2011_-challenged and uninfected horses, fold change was calculated (fold change: the normalised expression value of a gene in WNV-challenged horsecells/the normalised expression value of a gene in uninfected horse cells). **C**, **D** Immunolabeling of viral antigen and inflammatory cells in experimentally infected horse tissues. No viral antigen was detected (**A**) despite perivascular (arrow) and parenchymal infiltration of leukocytes, including CD3 + cells (**D**, red stain, arrow) in experimentally infected equine brain (Medulla oblongata). The results from horse no. 2 are presented and are representative of all three horses. Antigen of interest on both sections were visualised with AEC substrate (red product) and sections were counterstained with Meyer’s haematoxylin (Magnification: ×20).

To compare the magnitude of gene expression, the fold change was calculated. For this purpose, the delta delta Ct (∆∆Ct) values were calculated as follows: $\Delta \Delta {\text{Ct}} = \Delta {\text{Ct}}_{\text{WNV}} - \Delta {\text{Ct}}_{\text{mock}}$. The bar graphs in Figures 1, 2, 3, 4, 5, 6 and 7 and Additional file 1 show the expression of genes in WNV-stimulated PBMCs over mock-inoculated PBMCs (fold change: the normalised expression value of a gene in WNV-stimulated cells/the normalised expression value of a gene in mock-inoculated cells). Furthermore, accounting for the effects of culture conditions on gene transcription in WNV- and mock-inoculated equine PBMCs, relative expression of genes between WNV-stimulated $\left[ {\Delta \Delta {\text{Ct}}_{\text{WNV}} = \Delta {\text{Ct}}_{\text{WNV}} - \Delta {\text{Ct}}_{\text{fresh}} } \right]$ and mock-inoculated $\left[ {\Delta \Delta {\text{Ct}}_{\text{mock}} = \Delta {\text{Ct}}_{\text{mock}} - \Delta {\text{Ct}}_{\text{fresh}} } \right]$ cells were analysed. For this purpose, tocompare the normalised expression of genes from PBMCs harvested at each time point to their respective expression levels before either WNV- or mock- inoculation, the ∆∆Ct values were calculated by subtracting ∆Ct of genes in fresh-isolated PBMCs from the ∆Ct of genes in WNV- or mock- inoculated PBMCs at each time-point.

### Immunohistochemistry (IHC)

Immuno labeling for flavivirus NS1 antigen using the monoclonal antibody 4G4 has been previously described in [28]. Immunophenotyping of T-lymphocytes in inflammatory aggregates was achieved using cross-reactive anti-human CD3 (CD3 clone F7.2.38, Dako) mouse monoclonal antibody following previously described protocols [37]. For each IHC batch, a positive and negative antigen control was included.

### Virus growth kinetics quantification

The PBMCswere isolated from the three WNV_KUN_ seronegative horses, as described above. Cells were diluted to 1 × 10^5^ cells/mL in RPMI supplemented with 10% FBS. One mlaliquots of cells were pelleted by centrifugation, the supernatant discarded, and the cells resuspended with 100 µL medium containing 1 × 10^5^ PFU ofWNV_NSW201_ (MOI 1). The cells-virus suspension was incubated at 37 °C for 1 h for virus-adsorption. The suspensionswere agitated every 15 min. Each 100 µL of cell-virus suspension was then plated in one well of a 24-well cell culture plate, and 900 µL RPMI containing 10% FBS was added. The cells were incubated at 37 °C in a CO_2_ incubator until the designated time of harvest (day 1, 2, 3, and 4 pi). For the Vero cell positive control, DMEM with 5% FBS was used. Thermal-inactivation control involved addition of 1 × 10^5^ PFU/mL of WNV_NSW2011_ to wells without the presence of cells. These control wells underwent the same conditions as the test wells. A well for each of three horses, a Vero cell positive control and a thermal-inactivation control was harvested every 24 h for 4 days pi. For complete removal of adherent PBMCs, lidocaine was used [32], whereas trypsinization was performed for Vero cells. The virus concentration in supernatant was quantified using standard plaque assay [4].

### Detection of viral infectivity in PBMCs

The PBMCs harvested at 24 h pi were placed on two spots in circles drawn with a Dako pen on glass slides. The slides were air dried, formalin fixed and subjected to the immunolabelling protocol with monoclonal antibody 4G4 described above, except for omission of the deparaffinization steps.

### Statistical analysis

In general the technical replicates were averaged. The impact of virus challenge (treatment) and duration of incubation (time points) were evaluated using the SAS software package v. 9.2 (SAS institute, Cary, NC, USA). For this purpose, the GLM (general linear model; Proc GLM) procedure and the implemented analysis of variance (ANOVA) statistic were used. Pairwise comparisons were performed between the time points and treatment groups, using Tukey’s multiple comparisons in SAS, where *P* value was simultaneously adjusted. Besides, student’s *t* test was applied when treatment groups were compared. The data were expressed as means ± standard deviations (SD) of biological repeats and (*) *P* < 0.05 were set as statistically significant.

## Results

### Expression of interferon and cytokine genes

Expression of the mRNA for IFNα, β and γ were higher in WNV-stimulated PBMCs at most time points from 2 to 24 h compared to control PBMCs (Figure 1A). IFNα (Figure 1B) and IFNγ (Figure 1D) transcription were significantly upregulated at 6 h, whereas IFNβ (Figure 1C) transcription was significantly increased at 24 h pi. The expression of mRNA for cytokines IL1α, IL1β, IL12, and IL22 was increased at most time points in WNV-stimulated PBMCs compared to the mock-inoculated PBMCs (Additional file 1A). Pro-inflammatory cytokines IL6 and IL8 mRNA were increased 91 and 26 fold, respectively, at 24 h pi in virus induced PBMCs. IL22 mRNA expression was 75 fold higher in WNV-stimulated PBMCs compared to mock-inoculated PBMCs, whereas steady state expression of TNFα and IL12 mRNA was observed over times (Additional file 1A). Although the PTX3 gene transcript was upregulated in WNV-infected PBMCsat 2 h pi, it was down-regulated at the later hours pi (Additional file 1A). When IL1 gene transcription was compared between WNV-stimulated and mock-inoculated PBMCs, IL1α (Figure 2A) and IL1β (Figure 2B) transcription patterns were similar in both the stimulated and control cells. Notably, IL1α and IL1β mRNA expression level in both WNV-stimulated and mock-inoculated PBMCs were highest at 6 h pi (Figures 2A and B). IL6 (Figure 2C), 8 (Figure 2D), and 22 (Figure 2E) mRNA transcription were higher at 24 h pi in WNV-stimulated PBMCs compared to mock-inoculated PBMCs, whereas PTX3 mRNA (Figure 2F) was upregulated at 2 h pi in the virus-stimulated PBMCs.

### Expression of Toll-like receptors and associated downstream genes

The expression of TLR family (TLR1-10) gene transcripts was also quantified in WNV-infected equine PBMCs (Additional file 1B). TLR3, 5, 7, and 8 mRNA expression were increased early at 2 h pi, whereas TLR6 and 9 transcription levels were higher at 12 h pi. In addition to the higher expression at 2 h, TLR8 mRNA was increased at 24 h pi, whereas TLR1 mRNA was increased at 24 h pi only (Additional file 1B). When TLR3 (Figure 3A), TLR7 (Figure 3B) and TLR9 (Figure 3C) mRNA expression in WNV-stimulated PBMCs was compared with mock-inoculated PBMCs, TLR3 mRNA expression level was higher at 6 and 12 h pi, whereas TLR7 and 9 mRNA expression level were higher at 2 h pi. The time course expression of other TLRs in virus-infected and mock-inoculated cells are presented in Additional file 2. Transcripts of all the downstream genes associated with signalling of those TLRs quantified were up to four times upregulated in WNV-stimulated PBMCs compared to mock-inoculated PBMCs (Additional file 1C). TRAF3, IRF3, STAT1 and NF-κB mRNA showed similar patterns of expressions in PBMCs in response to WNV challenge. ThemRNA expression of these genes was upregulated at 2 h as well as 24 h pi (Additional file 1C). MyD88 (Figure 4A), TRAF3 (Figure 4B), STAT1 (Figure 4C), STAT2 (Figure 4D), IRF3 (Figure 4E), IRF7 (Figure 4F) and NF-κB (Figure 4G) mRNA were significantly upregulated in WNV-inoculated equine PBMCs compared to mock-inoculated PBMCs. Notably, STAT1 and 2 gene transcripts were upregulated from 2 h pi and during the remaining time of the experiment (Figures  4C and D). Beside TLRs-associated genes, the ISG15, SOCS1 and SOCS3 genes were also upregulated in horse PBMCsin response to WNV challenge (Figure 5A). When these gene transcription levels were compared between WNV-inoculated and mock-inoculated PBMCs, ISG15, SOCS1 and SOCS3 mRNA were up-regulated at 24 h pi in stimulated PBMCs (Figures 5B–D). Additionally, SOCS3 mRNA was upregulated at 2 h pi in WNV-inoculated PBMCs compared to mock-inoculated control cells (Figure 5D). Most of the gene transcription levels were found to be influenced by the time of stimulation and virus inoculation (Additional file 3).

### HMOX1 and caspase 3 genes expression

Transcriptomes related to oxidative stress (HMOX1) and apoptotic pathway (caspase 3) were quantified in equine PBMCs. Although there was steady state expression of HMOX1 mRNA levels, the caspase 3 mRNA level was increased in WNV-stimulated PBMCs when compared to mock-inoculated PBMCs (Figure 6A). Caspase 3 gene expression was significantly higher in virus-stimulated PBMCs at 12 h pi (Figure 6B).

### Quantification of selected gene transcripts and viral antigen detection in tissues from experimentally WNV_NSW2011_ infected horses

The mRNA expression patterns of selected genes (IFNα, β and γ, IL1β, IL22, PTX3, TNFα, ISG15, TLR3, TLR7, TLR9, MyD88, TRAF3, IRF7, STAT1 and 2) were also assessed in lymphoid tissues (spleen, pre-scapular lymph node draining the inoculation site) and brain (Medulla oblongata) from horses experimentally infected with WNV_NSW2011_ [50], terminated 12 days pi (Figure 7). While all these genes were transcribed in the lymphoid tissues, particularly high mRNA levels were seen for IFNγ, IL22, ISG15 and IRF7 (Figure 7A). In brain tissue, IFNα, CXCL10, TLR3, ISG15 and IRF7 mRNA expressions were also notably upregulated in the infected horses (Figure 7B). Notably, WNV_NSW2011_ RNA could not be detected in any tissues of infected horses (up to 40 cycles). Nor was viral antigen detectable in the brain of the three infected horses terminated at 12 days pi (Figure 7C) despite signs of inflammation, with CD3^+^ T-lymphocytes marginating blood vessels, and infiltrating the perivascular spaces and neuropil of the Medulla oblongata (Figure 7D).

### Virus growth kinetics in peripheral blood mononuclear cells

**Figure 8:**
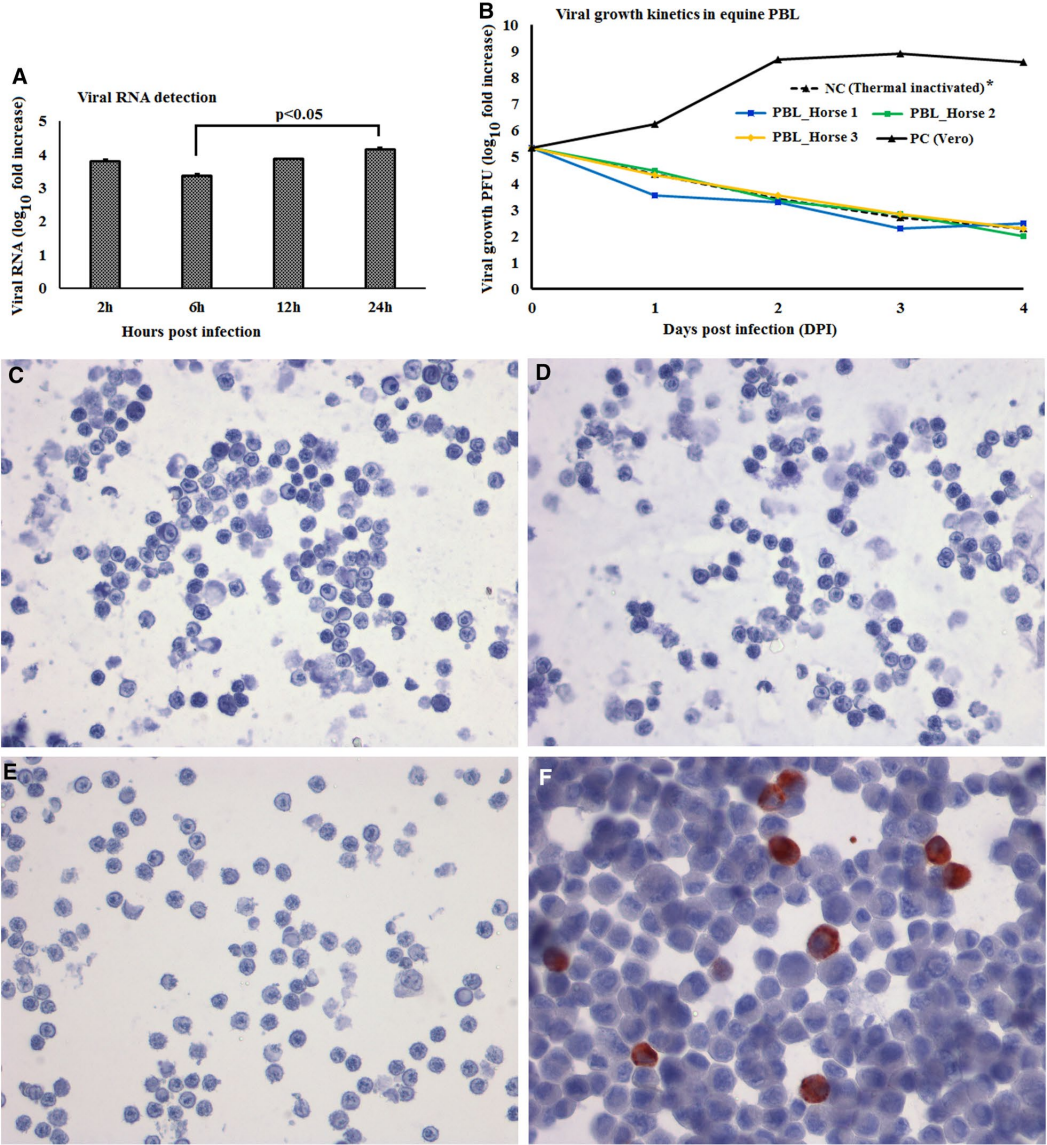
**Growth kinetics of WNV**_**NSW2011**_ **and detection of viral antigen in equine PBMCs in vitro.** **A** Viral RNA quantification in PBMCs using qRT-PCR, (**B**) Virus replication in cells quantified using plaque assay. *PC: positive control, NC: negative control. Thermal inactivated curve is superimposed by the other lines. **C** WNV-stimulated PBMCs immunostained using flavivirus NS1 specific monoclonal antibody 4G4, (**D**) WNV-stimulated PBMCs immunostained without 4G4, (**E**) Mock-stimulated PBMCs immunostained using 4G4 as negative control, and (**F**) WNV-stimulated Vero immunostained using 4G4 as positive control. (Magnification: ×20). Arrow, indicated the perivascular regions.

Viral RNA measurement showed higher levels of WNV_NSW2011_ RNA in WNV-inoculated equine PBMCs at 24 h pi compared to 6 h pi (Figure 8A). No viral RNA was detected in mock-inoculated equine PBMCs and Ct were set to 40 for delta Ct (ΔCt) calculation. In order to assess the permissiveness of equine PBMCs to WNV_NSW2011_ infection beyond the first 24 h, a 4-day (96 h) growth kinetics experiment was conducted. Virus titration by plaque assay was performed on PBMCs culture supernatant. The 96 h growth kinetics curve showed that the virus titres from wells with challenged PBMCs did not differ significantly from that of the thermal inactivation culture (cell-free culture control) (Figure 8B). This suggests that WNV_NSW2011_ did not productively replicate in equine PBMCs. The lack of NS1-specific immunolabeling of WNV_NSW2011_-challenged equine PBMCs on day 1 pi corroborated the failure of WNV_NSW2011_ to establish productive infection in equine PBMCs (Figure 8C). This contrasted with the high titres, as well as positive NS1-immunolabeling, observed in supernatants and cell pellet, respectively, from virus inoculated Vero cells (positive control) (Figures 8C and F). Mock-stimulated PBMCs immunostained using 4G4 as negative control are shown in Figure 8E.

## Discussion

Understanding the expression kinetics of genes involved in the innate immune recognition of virus is important for understanding the host-pathogen interactions and pathogenesis of disease. TLRs and associated downstream gene products are amongst the key molecules recognising virus and initiating the inflammatory cascades and antiviral responses immediately upon infections [38]. Blood peripheral leukocytes are thought to be amongst the first cells to come into contact with mosquito-inoculated WNV [27, 31] and thus PBMCs may act as the first line of defence against the virus. However, despite their potential importance, the interaction between WNV and equine PBMCs remains to be elucidated. As part of a greater aim to elucidate the genomic networks, this study identified the expression patterns of selected key genes in equine PBMCs following in vitro challenge with an equine-virulent Australian strain of WNV.

Among the ten members of the TLR family (TLR1-10), TLR3, 7, 8 and 9 recognise viral genomic components [39]. TLR3 has been associated with the direct recognition of double-stranded viral RNA, while TLR7 and TLR8 target single-stranded viral RNA [39]. The involvement of TLR3 and TLR7 has been extensively studied for WNV infection and recognition in mice [9, 10]. In this study, TLR1, 3, 5, 7 and 9 mRNA expressionsall became upregulated within 2–24 h of in vitro virus challenge of horse PBMCs suggestive of their involvement in the WNV-induced innate immune response in this species. MyD88 and NF-κB mRNA expression likewise became upregulated in WNV-exposed equine PBMCs at 2 h pi. The partial WNV-recognition pathway has been sketched in mouse [8, 40] where WNV is being sensed by TLR3 and 7. TLR3 and 7 lead to the activation of TRAF3 and MyD88, respectively. TRAF3 leads to the activation of IRF7, whereas IRF3 is activated by MyD88. IFN regulatory factors (IRFs) then lead to the production of IFNβ (reviewed in [8]). Subsequently, IFNβ and IFNα activate STAT1, STAT2 and IRF9, and these molecules then stimulate the transcription of antiviral genes such as the ISGs [8, 15]. The results of the present studies of equine PBMCs responses to WNV are in accordance with these pathways, with mRNA for TRAF3, IRF3, IRF7 and the STATs all being upregulated within 2 h post-challenge. While transcription of TLRs and associated genes were upregulated at early time points, ISG15 mRNA was upregulated at 24 h pi in the equine PBMCs in line with the time required to induce an antiviral state. This study identified higher expression of both Type-I (IFNα and IFNβ) and Type-II (IFNγ) IFNs in WNV-stimulated equine PBMCs compared to mock-inoculated PBMCs. The protective roles of IFNs in WNV infection have been widely studied in mice [41, 42]. Antiviral IFNs signal through the JAK/STAT pathway to induce ISG production. ISGs can also be directly induced by some IRFs in an IFN-independent pathway [43]. Either way, the ISGs function to block virus replication [43]. IFNβ gene expression was also reported to be upregulated in WNV_KUN_-induced human blood monocyte derived dendritic cells at 24 h pi and then decreased at 48 and 72 h pi [7]. It is important to note that phosphorylation of IRF3 in virus-infected cells can affect the virus-induced gene expression. TBK1 and IKKE are able to phosphorylate IRF3 and IRF7 in virus-infected cells, and can thereby control the IRF signalling pathway [44]. The phosphorylation related virus-induced down-stream genes expression has not been verified in this study. Furthermore, SOCS are the physiological suppressors of cytokine signalling and form part of a classical feedback loop. SOCS1 and SOCS3 siRNA treated macrophages facilitated higher replication of WNV in the mouse [15] and up-regulation of SOCS1 and SOCS3 mRNA has been found in mouse in response to WNV infection [45]. Higher expressions of SOCS1 and 3 at 2 h pi may contribute to the restriction of WNV replication and balancing the immune responses of equine PBMCs in response to WNV_NSW2011_.

The present study involved infection of equine PBMCs with WNV_NSW2011_ at MOI 1. Styer et al. [31] reported that mosquitoes typically inoculate ~10^2^ PFU directly into the blood while probing and feeding and thus circulating PBMCs are amongst the first cells to be exposed to WNV. We found that higher viral RNA was detected at 24 h pi compared to 6 h pi in equine PBMCs. Therefore, an extended experiment up to 4 days pi was conducted, in order to investigate whether WNV_NSW2011_ can productively replicate in equine PBMCs. It is important to note that for successful replication, viruses will required several hours to complete the replication cycle, which includes production of mature virions and subsequently exocytosis from cells as infectious viral particles [46]. Following a mosquito bite, the initial WNV infection and spread (the early phase) and peripheral viral amplification (the visceral-organ dissemination phase) may take up to 4 days [40]. Therefore, virus was not quantified using plaque assay in this study until 24 h pi and later. Interestingly, we found that WNV_NSW2011_ did not replicate in the equine PBMCs, which was further supported by the lack of expression of viral NS1 at 24 h pi. Although in vitro WNV inoculation by passes the host-virus interfaces in the skin, the host-viral interactions of virus delivered into the blood might be represented by the type of study employed here. In this context, the higher expression of Type I IFNs and ISG15 in the first 24 h pi may explain the inhibition of viral replication or growth in equine PBMCs. These early virus restriction mechanisms may point to a natural resistance mechanism(s) in this species and are in accordance with the fact that the majority of infections in horses are subclinical [1].

Rawle et al. [7] compared the growth kinetics of WNV_NSW2011_ and WNV_NY99_ using plaque assay and found that WNV_NSW2011_ did not replicate in human blood monocyte derived dendritic cells as they extended the experiment from 24 to 72 h pi, whereas WNV_NY99_ successfully replicated in these cells for the duration of the experiment. The successful replication of WNV_NY99_ has also been reported in equine PBMCs [27], where in vitro equine PBMCs were infected for 10–14 days pi. Since other studies [7, 27] did not quantify viral RNA during the first 24 h pi, a comparison to our findings of viral RNA detection is precluded. Our results of WNV_NSW2011_ growth kinetics, quantified using plaque assay, corroborates the growth kinetics of this virus strain reported by Rawle et al. [7] but differs from the growth kinetics of WNV_NY99_ reported by Rawle et al. [7] and Gracia-Tapia et al. [27], respectively. Whether these differences in growth kinetics in leukocytes between WNV_NSW2011_ and WNV_NY99_ maybe ascribed to virus characteristics sensu stricto or may be explained by their respective ability to induce protective innate immune responses remains to be elucidated.

Other than IFNs (both Type I and Type II), transcripts for several cytokines were found to be upregulated in response to WNV stimulation of equine PBMCs, including IL1α, IL1β, IL6 and IL8. Studies with IL1R1 knockout mice have demonstrated that IL1 is critical for limiting WNV-induced encephalitis in the murine model, purportedly via a T cell activation mechanism [47] and mice inoculated intraperitoneally with WNV_NY99_ had upregulation of IL6 and IL12 transcripts [48]. It is important to note that when traditional fold changes are calculated to demonstrate gene expression, the culture conditions may confound the results. Therefore, in addition to the traditional method (Figures 1A, 5A, and 6A; Additional file 1), we compared the normalized expression of cytokine genes from PBLs harvested at each time point to their respective expression levels before either WNV or mock inoculation (Figures 1B–D, 2A–F, 3A–C, 4A–G, 5B–D, 6B; Additional file 2). This method display the temporal kinetics of cytokine gene transcription in mock-inoculated PBLs, which in turn can reveal background effects of culture conditions on gene transcription in the WNV-challenged PBLs. Using this approach, we demonstrated that IL1α, IL1β and IL6 mRNA expression levels were increased in both sets of culture conditions.

A holistic transcriptome expression study using microarray found higher expression of IL12, IL22, SOCS3 and PTX3 in the thalamus of experimentally WNV_NY99_-infected horses [18]. Similar to the in vitro experiment, we found that transcription of selected cytokine genes was upregulated in brain, spleen and draining lymph node of experimentally WNV_NSW2011_-infected horses. We found that while IFNγ and IL22 mRNA expression level was higher in lymphoid tissues (lymph node and spleen), their expression level was lower in CNS in experimentally WNV_NSW2011_-infected horse. It has been suggested that IL22 exacerbates lethal WNV_NY99_ encephalitis by promoting virus neuro invasion in mice [49]. Therefore, lower expression of IL22 and higher expression of antiviral IFNα in equine brain at 12 days pi may explain the absence of viral antigen in the brain of experimentally infected horse in this study. Notably, higher expression of IL22 in experimentally infected horse tissues (spleenand lymph node) coincided with the expression of this gene in experimentally infected horse thalamus [18] implying that species is also an important factor to be considered. Furthermore, despite mild inflammations in the CNS, no viral antigen was detected by IHC and qRT-PCR, suggesting virus clearance by at least 12 days pi. Similar results have been demonstrated in other studies in both experimentally and naturally infected horses [20, 21, 50]. It remains to be established exactly what role each individual cytokine plays in virus control and inflammation in equine WNV encephalitis, but the prominent transcription of the chemokine ligand CXCL10 in the experimentally infected equine brain may at least explain the CD3^+^ cell infiltrates in the brain.

Caspase 3 mRNA expression was increased over time in equine PBMCs in response to in vitro WNV_NSW2011_. WNV has been reported to induce apoptosis in human brain derived glia cells in culture by the activation of caspase 3, 8 and 9 [51] but so far there has been no study that quantified caspase expression in equine PBMCs following WNV challenge. Higher expression of caspase 3 mRNA in in vitro infected equine PBMCs is in accordance with other studies [51, 52] establishing that caspase 3-dependent apoptosis might be involved in WNV infection. It remains to be shown that the equine cells proceed to undergo apoptosis following WNV exposure in vitro and in vivo. On the other hand, comparatively lower expression of TNFα and HMOX1 mRNA in this study suggests that oxidative stress may not be important in WNV_NSW2011_ induced early immune responses in equine PBMCs.

Mosquito-borne diseases are a major human and animal health issue globally as well as in Australia. The socioeconomic importance of these diseases to the equine industries is substantial and their impact is predicted to increase in some regions due to changes in vector breeding associated with climate change. This has been well demonstrated by the unprecedented outbreak of WNV encephalitis in horses in SE Australia in 2011 following flooding of many eastern regions of Australia. Although the expression of most of the genes studied here was similar to those found in the mouse model, the murine immune response to WNV may not necessarily reflect the immune responses in human and horse [19, 53]. Therefore, studies in horse PBMCs may provide a better understanding of the early interactions between host cells and viruses, at least as far as horses are concerned.The expression kinetics of the selected transcriptomes illustrates a partial pathway by which WNV is detected by the host immune system. Further studies will be required to elucidate the complete network of antiviral defences.

## Additional files

10.1186/s13567-016-0347-8 **Expression kinetics of cytokines, TLRs and TLR-associatedgenes in equine PBMCs in response to West Nile virus in fold change.** A) Cytokines expression in fold change. The ∆∆Ct [∆∆Ct = ∆Ct_WNV_ - ∆Ct_mock_] values were calculated by subtracting the ∆Ct of genes in mock-inoculated PBMCs. The bar graph showed the expression of genes in WNV-infected PBMCs over mock-inoculated PBMCs (fold change: the normalised expression value of a gene in WNV-stimulated cells / the normalised expression value of a gene in mock-inoculated cells). Bars without common superscripts (A, B) denote statistical significant difference among time points (*P* < 0.05). B) Toll-like receptors expression in fold change. The ∆∆Ct [∆∆Ct = ∆Ct_WNV_ - ∆Ct_mock_] values were calculated by subtracting the ∆Ct of genes in mock-inoculated PBMCs. The bar graph showed the expression of genes in WNV-infected PBMCs over mock-inoculated PBMCs (fold change: the normalised expression value of a gene in WNV-stimulated cells / the normalised expression value of a gene in mock-inoculated cells). Bars without common superscripts (A, B) denote statistical significant difference among time points (*P* < 0.05). C) TLRs-associated downstream genes expression in fold change. The ∆∆Ct [∆∆Ct = ∆Ct_WNV_ - ∆Ct_mock_] values were calculated by subtracting the ∆Ct of genes in mock-inoculated PBMCs. The bar graph showed the expression of genes in WNV-infected PBMCs over mock-inoculated PBMCs (fold change: the normalised expression value of a gene in WNV-stimulated cells / the normalised expression value of a gene in mock-inoculated cells). Bars without common superscripts (A, B) denote statistical significant difference among time points (*P* < 0.05).
10.1186/s13567-016-0347-8 **Time-depended relative expression of TLRs mRNAs in equine PBMCs in response to West Nile virus.** Description of data: Relative expression of TLRs mRNA, accounting for the effects of culture conditions on gene transcription in WNV- and mock-inoculated equine PBMCs. To compare the normalised expression of TLRs genes from PBMCs harvested at each time point to their respective expression levels before either WNV- or mock- inoculation, the ∆∆Ct values were calculated by subtracting ∆Ct of genes in fresh-isolated PBMCs from the ∆Ct of genes in WNV- or mock- inoculated PBMCs at each time-point (for WNV-stimulated PBMCs, ∆∆Ct_WNV_ = ∆Ct_WNV_ - ∆Ct_fresh_; and for mock-inoculated PBMCs, ∆∆Ct_mock_ = ∆Ct_mock_ - ∆Ct_fresh_). **P*<0.05.
10.1186/s13567-016-0347-8 **Effect of time and virus stimulation on the relative expression of genes.** Summary of the Proc GLM (SAS) analysis detecting effect of time and virus stimulation on the mRNA expressions level of genes. Adjusted *P*-values are mentioned in the columns for time of WNV-inoculation, WNV-stimulation, and the interaction of incubation time and virus stimulation.
