# Supplementary figures and images for: Kinetics of the West Nile virus induced transcripts of selected cytokines and Toll-like receptors in equine peripheral blood mononuclear cells
Source: Vet Res. 2016 Jun 7;47:61. doi: 10.1186/s13567-016-0347-8 (PMC4895877; doi:10.1186/s13567-016-0347-8)

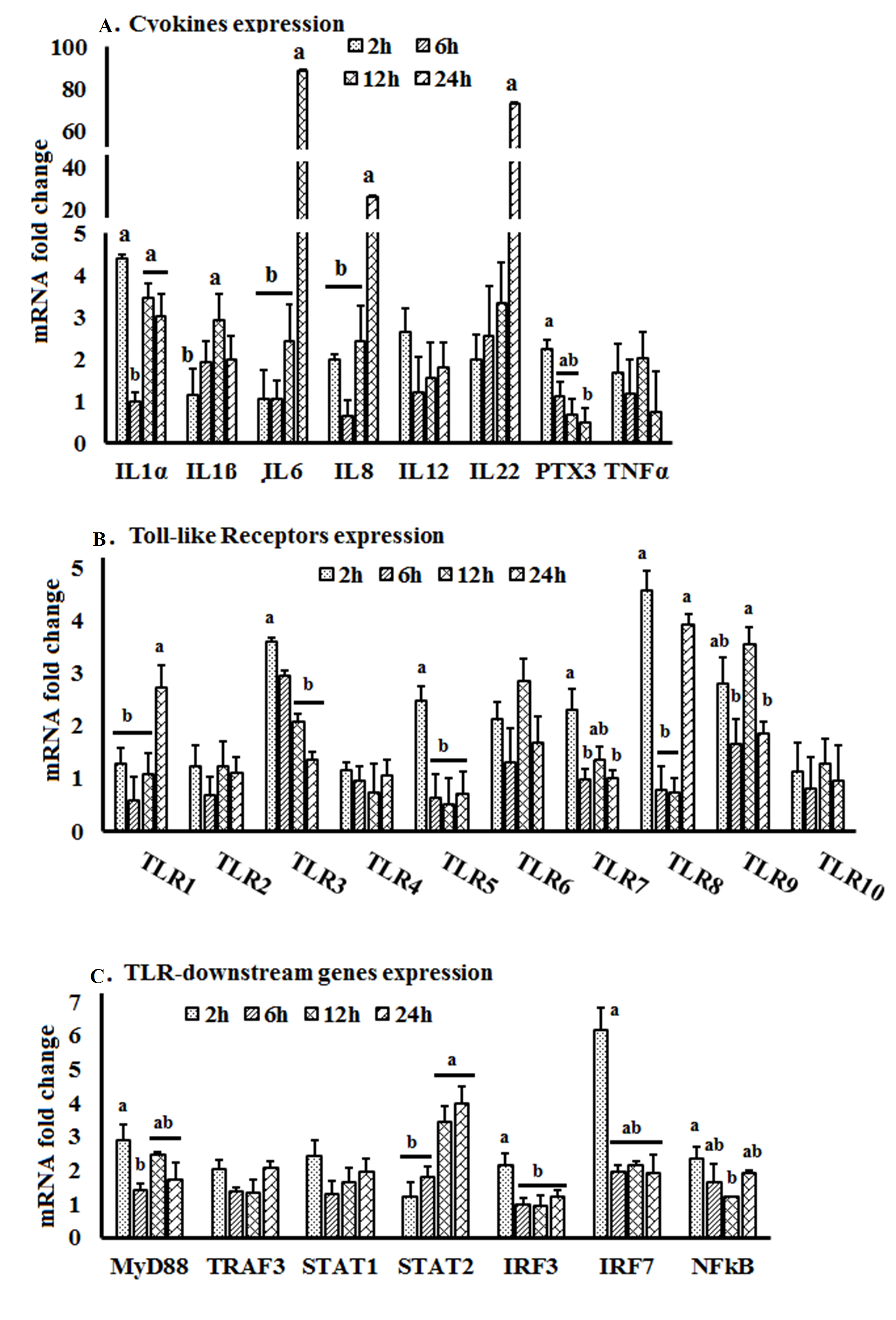

Supplement: Supplementary file 1 — 10.1186/s13567-016-0347-8 Expression kinetics of cytokines, TLRs and TLR-associatedgenes in equine PBMCs in response to West Nile virus in fold change. A) Cytokines expression in fold change. The ∆∆Ct [∆∆Ct = ∆CtWNV - ∆Ctmock] values were calculated by subtracting the ∆Ct of genes in mock-inoculated PBMCs. The bar graph showed the expression of genes in WNV-infected PBMCs over mock-inoculated PBMCs (fold change: the normalised expression value of a gene in WNV-stimulated cells / the normalised expression value of a gene in mock-inoculated cells). Bars without common superscripts (A, B) denote statistical significant difference among time points (P < 0.05). B) Toll-like receptors expression in fold change. The ∆∆Ct [∆∆Ct = ∆CtWNV - ∆Ctmock] values were calculated by subtracting the ∆Ct of genes in mock-inoculated PBMCs. The bar graph showed the expression of genes in WNV-infected PBMCs over mock-inoculated PBMCs (fold change: the normalised expression value of a gene in WNV-stimulated cells / the normalised expression value of a gene in mock-inoculated cells). Bars without common superscripts (A, B) denote statistical significant difference among time points (P < 0.05). C) TLRs-associated downstream genes expression in fold change. The ∆∆Ct [∆∆Ct = ∆CtWNV - ∆Ctmock] values were calculated by subtracting the ∆Ct of genes in mock-inoculated PBMCs. The bar graph showed the expression of genes in WNV-infected PBMCs over mock-inoculated PBMCs (fold change: the normalised expression value of a gene in WNV-stimulated cells / the normalised expression value of a gene in mock-inoculated cells). Bars without common superscripts (A, B) denote statistical significant difference among time points (P < 0.05). [file 13567_2016_347_MOESM1_ESM.tif]

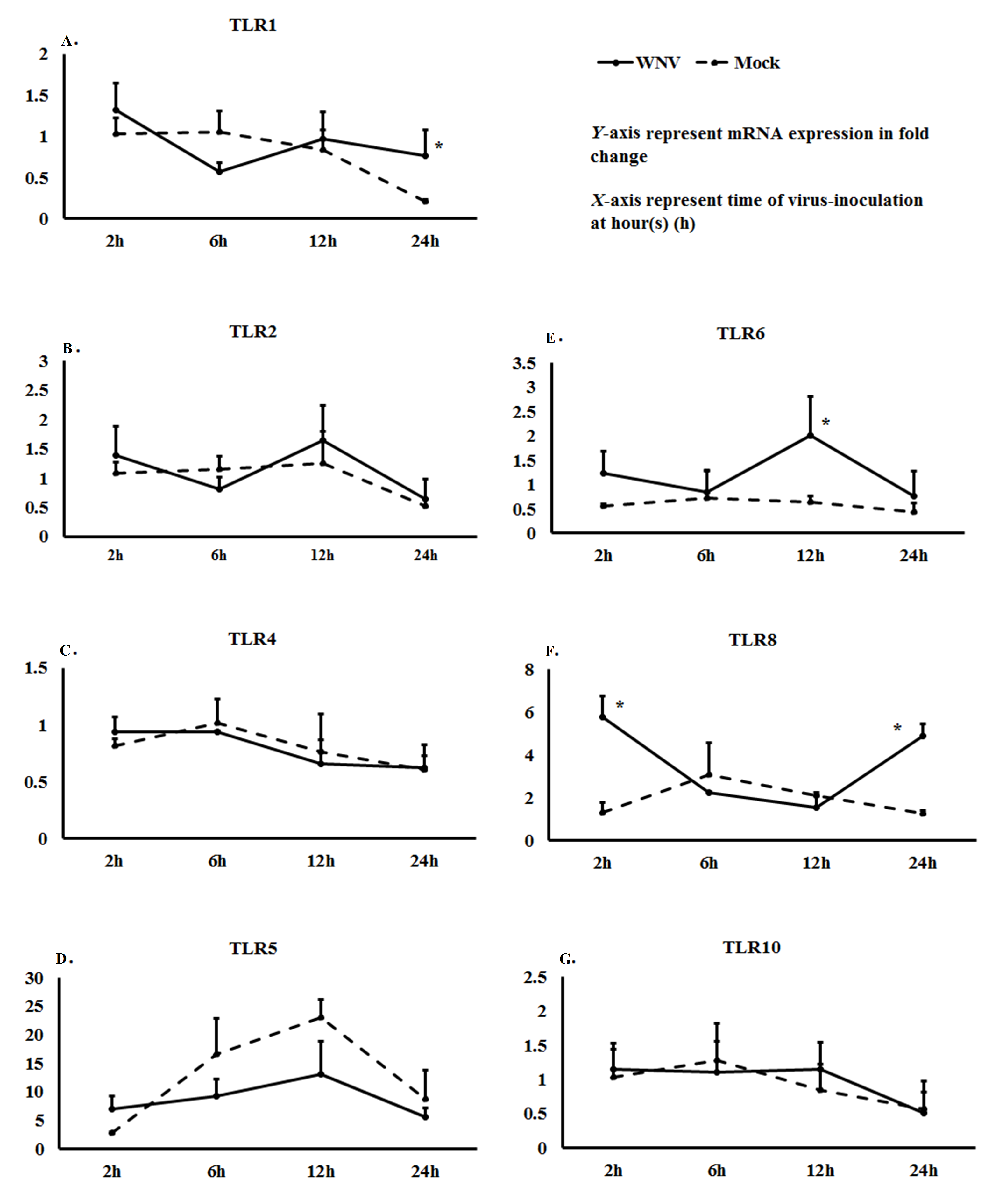

Supplement: Supplementary file 2 — 10.1186/s13567-016-0347-8 Time-depended relative expression of TLRs mRNAs in equine PBMCs in response to West Nile virus. Description of data: Relative expression of TLRs mRNA, accounting for the effects of culture conditions on gene transcription in WNV- and mock-inoculated equine PBMCs. To compare the normalised expression of TLRs genes from PBMCs harvested at each time point to their respective expression levels before either WNV- or mock- inoculation, the ∆∆Ct values were calculated by subtracting ∆Ct of genes in fresh-isolated PBMCs from the ∆Ct of genes in WNV- or mock- inoculated PBMCs at each time-point (for WNV-stimulated PBMCs, ∆∆CtWNV = ∆CtWNV - ∆Ctfresh; and for mock-inoculated PBMCs, ∆∆Ctmock = ∆Ctmock - ∆Ctfresh). *P<0.05. [file 13567_2016_347_MOESM2_ESM.tif]
