# Supplementary material for: Kinetics of the West Nile virus induced transcripts of selected cytokines and Toll-like receptors in equine peripheral blood mononuclear cells
Source: Vet Res. 2016 Jun 7;47:61. doi: 10.1186/s13567-016-0347-8 (PMC4895877; doi:10.1186/s13567-016-0347-8)
Supplement: Supplementary file 3 — 10.1186/s13567-016-0347-8 Effect of time and virus stimulation on the relative expression of genes. Summary of the Proc GLM (SAS) analysis detecting effect of time and virus stimulation on the mRNA expressions level of genes. Adjusted P-values are mentioned in the columns for time of WNV-inoculation, WNV-stimulation, and the interaction of incubation time and virus stimulation. [file 13567_2016_347_MOESM3_ESM.docx]

| Gene | Time of WNV-inoculation | WNV-stimulation | Time*stimulation | R^2^ | Model |
| --- | --- | --- | --- | --- | --- |
| IFNα | 0.0002 | <0.0001 | 0.0315 | 0.485 | <0.0001 |
| IFNβ | <0.0001 | <0.0001 | 0.0187 | 0.895 | <0.0001 |
| IFNγ | 0.011 | 0.009 | 0.89 | 0.603 | 0.0018 |
| TNFα | 0.002 | 0.27 | 0.47 | 0.626 | 0.012 |
| IL1α | <0.0001 | <0.0001 | 0.0003 | 0.97 | <0.0001 |
| IL1β | <0.0001 | 0.0098 | 0.508 | 0.788 | 0.0002 |
| IL6 | 0.0002 | <0.0001 | <0.0001 | 0.882 | <0.0001 |
| IL8 | 0.0003 | <0.0001 | <0.0001 | 0.884 | <0.0001 |
| IL12 | 0.3 | 0.09 | 0.82 | 0.329 | 0.3969 |
| IL22 | <0.0001 | <0.0001 | 0.0005 | 0.884 | <0.0001 |
| PTX3 | 0.012 | 0.596 | 0.0009 | 0.727 | 0.0014 |
| TLR1 | 0.0015 | 0.283 | 0.043 | 0.694 | 0.0031 |
| TLR2 | 0.0051 | 0.836 | 0.508 | 0.570 | 0.0310 |
| TLR3 | <0.0001 | <0.0001 | <0.0001 | 0.986 | <0.0001 |
| TLR4 | 0.063 | 0.770 | 0.561 | 0.400 | 0.228 |
| TLR5 | 0.0004 | 0.458 | 0.029 | 0.736 | 0.0011 |
| TLR6 | 0.172 | 0.009 | 0.644 | 0499 | 0.0810 |
| TLR7 | <0.0001 | 0.008 | 0.017 | 0.817 | <0.0001 |
| TLR8 | 0.095 | 0.0001 | <0.0001 | 0.847 | <0.0001 |
| TLR9 | 0.013 | 0.001 | 0.5767 | 0.668 | 0.0054 |
| TLR10 | 0.075 | 0.89 | 0.902 | 0.357 | 0.3250 |
| MyD88 | 0.014 | <0.0001 | 0.1755 | 0.776 | 0.0003 |
| TRAF3 | 0.011 | <0.0001 | 0.084 | 0.818 | <0.0001 |
| STST1 | 0.0005 | 0.0014 | 0.494 | 0.733 | 0.0006 |
| STST2 | <0.0001 | 0.0001 | 0.0840 | 0.854 | <0.0001 |
| IRF3 | 0.0002 | 0.0079 | 0.0048 | 0.799 | 0.0001 |
| IRF7 | 0.0001 | <0.0001 | 0.023 | 0.8668 | <0.0001 |
| NF-κB | <0.0001 | <0.0001 | 0.1068 | 0.865 | <0.0001 |
| ISG15 | <0.0001 | 0.0013 | 0.0010 | 0.892 | <0.0001 |
| SOCS1 | 0.570 | 0.004 | 0.099 | 0.561 | 0.035 |
| SOCS3 | 0.0392 | <0.0001 | 0.0016 | 0.823 | <0.0001 |
| Caspase 3 | 0.174 | 0.006 | 0.0496 | 0.6122 | 0.015 |
| HMOX1 | 0.757 | 0.217 | 0.955 | 0.164 | 0.8553 |
